# Giant Hepatic Hemangioma Regressed Significantly Without Surgical Management: A Case Report and Literature Review

**DOI:** 10.3389/fmed.2021.712324

**Published:** 2021-08-19

**Authors:** Jingcong Zhang, Zuyang Ye, Lei Tan, Jinmei Luo

**Affiliations:** ^1^Department of Internal Medicine, Medical Intensive Care Unit and Division of Respiratory Diseases, Third Affiliated Hospital of Sun Yat-sen University, Guangzhou, China; ^2^Department of Nephrology, The Second Affiliated Hospital of Guilin Medical University, Guilin, China; ^3^Department of Medical Ultrasonic, Third Affiliated Hospital of Sun Yat-sen University, Guangzhou, China

**Keywords:** giant hepatic hemangioma, vascular endothelial growth factor, azithromycin, case repot, congenital vascular anomalies

## Abstract

Hepatic hemangioma (HH) is a congenital vascular anomaly comprising networks of abnormal blood and/or lymphatic vessels with endothelial cell proliferation. Their pathophysiology is not fully understood, and no specific drug is available to treat them. Conservative management, which limits observation, is preferred for most patients. A HH larger than 4 cm is considered a giant HH that may be treated using surgery ranging from embolization to hepatic resection or liver transplantation. Here, we describe a case with multiple and giant HHs that regressed significantly after treatment with azithromycin (AZM). A systematic literature review of HH and the effects of AZM on angiogenesis was then conducted.

## Background

HH is the most common benign vascular tumor, accounting for ~70% of all benign liver lesions; additionally, it is found in 0.7–7% of the general population and is often discovered incidentally on abdominal imaging performed for other indications (1, 2). Recently, with the development of imaging technology, HH can be diagnosed clearly and reliably using ultrasound (US)/computed tomography (CT) and magnetic resonance imaging (MRI) (3–7). HH usually presents in adulthood, with the average age of diagnosis varying from 30 to 50 years (8). No specific drug is available to treat HH, and the surgical approaches for HH range from embolization to hepatic resection or liver transplantation (LT) (9).

HH and infantile hemangioma (IH) are both congenital vascular anomalies. Although no specific drug is available for HH, some drugs, such as bleomycin, interferon-alpha, vincristine, cyclophosphamide, corticosteroids, β-blockers and sirolimus, induce IH regression, though their specific mechanisms in vascular malformations remain unclear (10–16). Corticosteroids, which dramatically block the vasculogenic potential of HemSCs by directly inhibiting VEGF-A expression, are master regulators of angiogenesis and vasculogenesis (17–19). Propranolol, a β-blocker, suppresses the vasculogenic potential of HemSCs by reducing cAMP levels and simultaneously activating the mitogen-activated protein kinase (MAPK)-extracellular signal-regulated kinase (ERK) pathway (20). Sirolimus (rapamycin), an antifungal, antineoplastic, antibacterial macrolide drug and the most studied compound to treat vascular anomalies, directly targets the RAS-MAPK-ERK and phosphatidylinositol 3 kinase (PIK3)/protein kinase B/mammalian target of rapamycin (mTOR) pathways in vascular malformations (4, 21–24). Everolimus, an mTOR inhibitor derived from sirolimus, has also been used sporadically to treat vascular malformations (4). Levy et al. reported a case of diffuse HHs successfully treated using sirolimus and high-dose propranolol (11).

AZM is a widely used macrolide antibiotic with high tissue permeability and cell adhesion. It is a Food and Drug Administration-approved antibiotic and the primary drug to treat chlamydia and mycoplasma pneumonia. In addition to its antimicrobial activity, AZM also exerts anti-inflammatory and antiproliferative effects (25, 26). AZM inhibits tumor angiogenesis by attenuating VEGF (27). However, the effect of AZM on hemangioma has not yet been reported.

Here, we describe a patient whose HHs regressed significantly without surgical management because of AZM.

## Case Presentation

A 26-year-old Chinese man who was previously healthy and had no significant medical or family history of any significant disease incidentally found right hepatic masses on ultrasonography (without detail and images) at the local hospital in March 2013. Among these masses, the largest one was 75 × 61 mm in size. At that time, the patient did not have any discomfort, such as abdominal distension or abdominal pain, and his laboratory values and serum tumor markers were all within normal limits. During the next 12 months, he did not complain of discomfort or take any medication.

**Figure 1:**
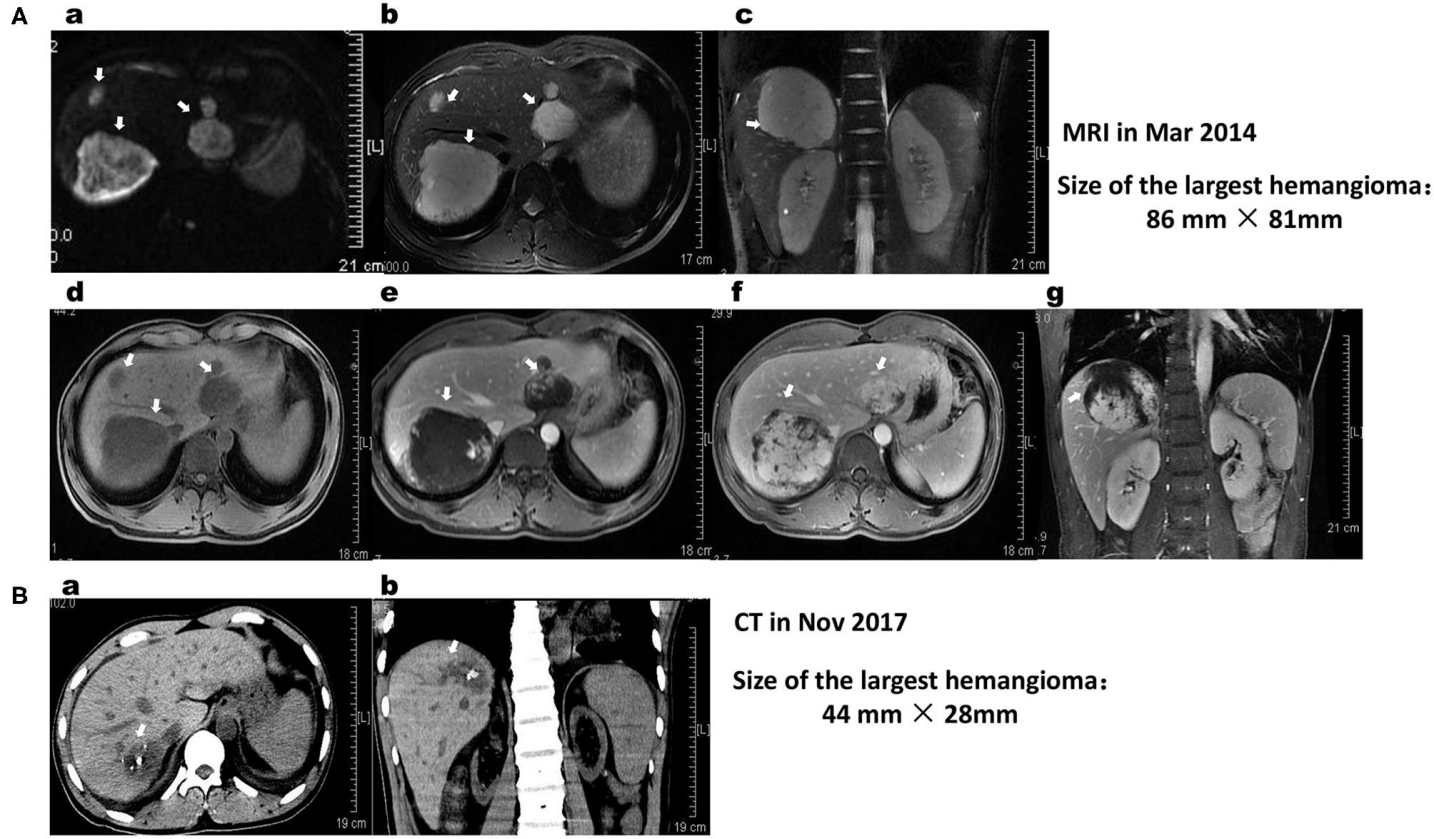
**(A)** MRI of the liver shows lobulated lesions in the subcapsular aspect of segments 2, 7, and 8 of the liver (white arrows). The largest lesion is located at segment 7, its size is ~86 × 81 × 81 mm, and it is closely related to the liver segments of the right hepatic vein and IVC. The size of the lesion in S2 is ~54 × 44 × 41 mm. The hemangiomas on MRI appear hyperintense on DWI (a) and T2WI (b,c) and hypointense on T1WI (d–g) sequences. On post-contrast dynamic imaging, the lesion demonstrates discontinuous peripheral nodular enhancement on the arterial phase (e) with incomplete centripetal fill-in on the delayed phase (d,f). The findings are consistent with hepatic hemangiomas. **(B)** Comparing the cross-sectional CT (a) and coronal (b) reformatted CT images with those of MRI images on March 16, 2014, primary multiple hemangiomas in the liver were significantly shrunk, and their boundaries were not clear. The size of the giant hemangioma in segment 7 of the liver was decreased to 44 × 28 mm, with unclear borders and nodular high-density shadows inside it. The lesions are closely related to the liver segments of the right hepatic vein and IVC.

In March 2014, to further diagnose the liver lesions, he visited our hospital. On admission, the patient's physical examination, laboratory indicators, including routine blood tests, routine urine tests, liver function, kidney function, coagulation function (FDP 2.34 mg/L), AFP, CEA, tumor-associated carbohydrate antigens (CA125 and CA199) and rheumatism-related indicators, were all within the normal limits. Abdominal MRI showed well-defined, smooth edges and homogeneous, solid, round-like lesions originating from the second to eighth segments of the right lobe of the liver (Figure 1A). The lesions displayed hypointensity on T1-weighted sequences (T1WI), hyperintensity on T2-weighted sequences (T2WI) and diffusion-weighted images (DWI), peripheral enhancement in the arterial phase and contrast retention in delayed phases. The largest mass in liver segment 7 was ~86 × 81 × 81 mm in size and was closely related to the right hepatic vein and inferior vena cava (IVC). Despite the large mass size, the hepatic vein and IVC were not compressed. He was diagnosed with giant HHs, and transarterial embolization or radiofrequency ablation was suggested. However, the patient rejected this advice for the time being. From April 2014, he started to cough and had throat pain, which was relieved after drinking a large amount of water and resting but often recurred. He thought the discomfort was related to his teaching career and did not seek medical help. In August 2014, he visited our hospital because of his HHs. However, he did not receive interventional treatment for the positive findings of urine protein and urine occult blood, as well as an increased level of serum creatinine to 314 μmol/L. He then visited another hospital and had undergone a histopathological examination of percutaneous renal biopsy. He was diagnosed with IgA nephropathy, chronic kidney disease (stage 4), secondary hypertension, chronic tonsillitis and HHs and was treated with amlodipine besylate, metoprolol, furosemide, and terazosin hydrochloride. Under the above treatments, his renal function did not improve but deteriorated progressively; thus, he started regular dialysis 3 times a week in October 2014.

**Figure 2:**
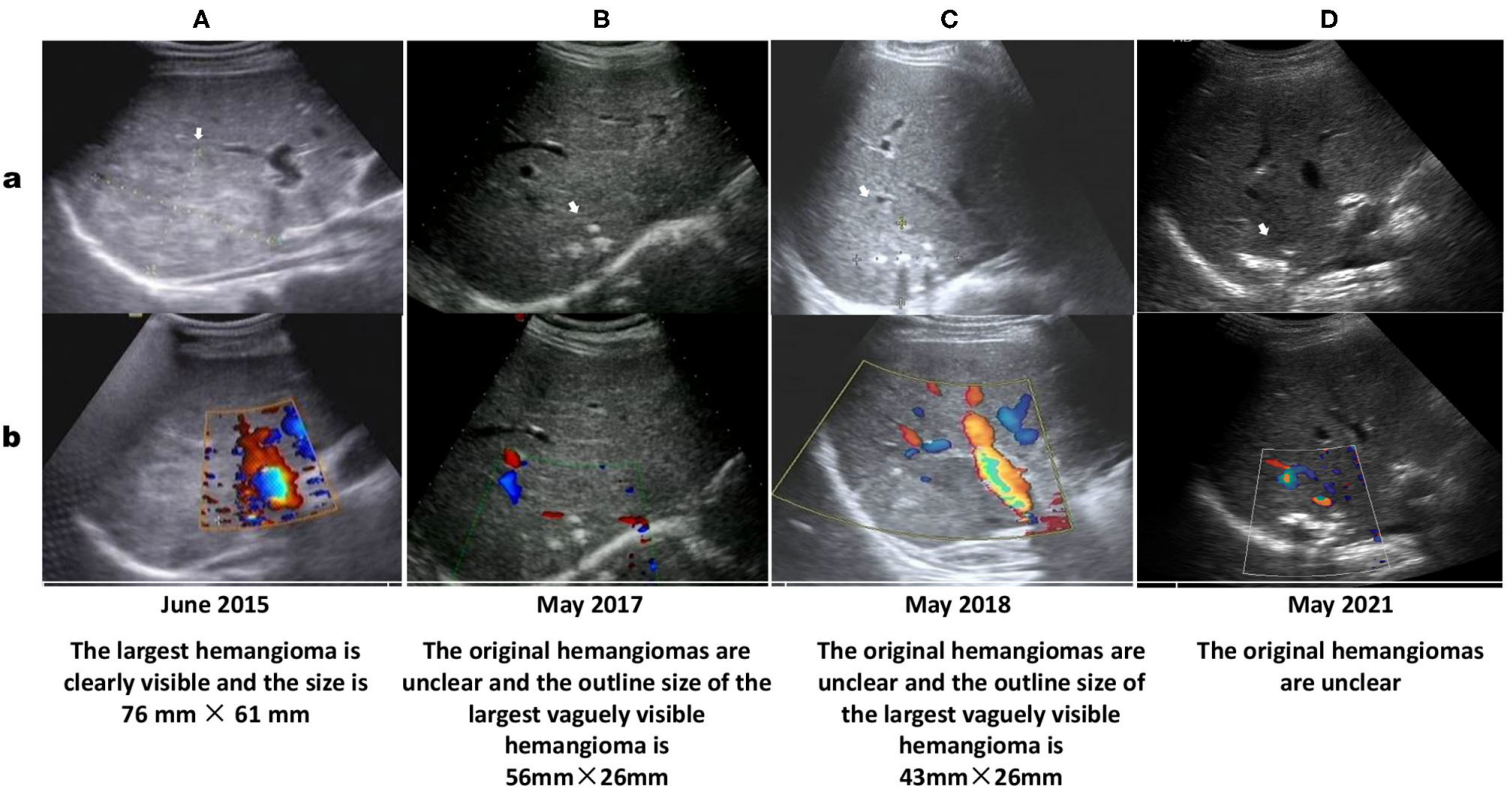
Ultrasound showing multiple hemangiomas (white arrows) that appear as echogenic nodular lesions on grayscale ultrasound (a) and non-vascular lesions on color Doppler ultrasound (b). The size of the largest segment located in the S7 segment on US performed in June 2015 (**A**: 76 × 61 mm) is nearly as large as that on US performed in March 2013 (according to the US report without images, 75 × 62 mm). Ultrasound images in May 2017 **(B)**, May 2018 **(C)** and May 2021 **(D)** show that most of the multiple hepatic hemangiomas are not clearly displayed, the largest hemangioma in the S7 segment is still visible (The size described in ultrasound report performed in May 2018 is 43 × 26 mm), and calcified plaques can be seen inside.

In June 2015, he was hospitalized in the ICU at the local hospital because of acute heart failure. During hospitalization, ultrasound examination reported multiple hemangiomas in his liver, and the largest hemangioma located in segment 7 was 76 × 61 mm in size (Figure 2A).

In December 2016, he was prescribed AZM tablet 0.5 qd for 5 days and then oral 0.25 qod for the long term to treat his recurrent tonsillitis and regulate his immunity. His cough was relieved, and his tonsillitis was cured.

In May 2017, Doppler ultrasound showed that his HHs were significantly smaller than before (56 mm × 26 m vs. 76 × 61 mm), with unclear outlines and shapes, strong echo points and normal internal blood vessels inside the lesions (Figure 2B). In November 2017, abdominal CT before kidney transplant surgery showed that the original multiple HHs in his right liver were blurred in outline and shape, and the size of the largest HH was reduced to 44 mm × 28 m, with an obscure outline, uneven internal echo, normal internal blood vessels, and plaque-like hyperechoic shadows behind it (Figure 1B).

Follow-up of the patient by phone during over 3 years indicated that the patient regularly took tacrolimus, mycophenolate mofetil, methylprednisolone, amlodipine besylate, metoprolol and terazosin hydrochloride. His blood pressure was controlled at a normal level, and his liver and kidney functions were normal with no anemia, hematuria or proteinuria. Results of abdominal ultrasound examination in May 2017 (Figure 2C) and May 2018 (Figure 2D) reported that the original HHs in the patient's liver were unclear.

## Discussion

HHs, the most common benign tumors of the liver, are congenital vascular anomalies without malignant potential based on current evidence (4, 28). Most HHs are found in women within the fourth and fifth decade of life and often originate from the right hepatic lobe (23, 28). Typical HHs can be diagnosed straightforward by a characteristic imaging appearance, with the reported diagnostic sensitivity and specificity of ultrasound (US), computed tomography (CT), and magnetic resonance imaging (MRI) for HHs being 96.9% and 60.3, 98.3% and 55, 98, and 99%, respectively (5, 16). The unique imaging features of HHs are the presence of peripheral nodular enhancement and progressive centripetal fill-in (5, 16). Important ultrasonographic findings of HHs are characterized as the absence of a lateral shadow (100%) and no attenuation of posterior echoes (100%), while the presence of a hyperechoic rim is useful for detecting isoechoic hemangioma (29). On CT, HHs are described as typical well-demarcated hypodense masses with peripheral nodular enhancement and centripetal homogeneous filling or atypical patterns with cystic areas, fibrosis or thrombosis (3, 5, 30). On MRI, HHs have similar images as those on CT, with classic hypointensity on T1-weighted sequences (T1WIs) and intense bright intensity on T2-weighted (T2WI) and diffusion-weighted images (DWIs) (5, 30). In this patient, the characteristic abdominal MRI scan (Figure 1A), CT scan (Figure 1B) and Doppler ultrasound examinations (Figure 2) all confirmed that the multiple masses in his liver were HHs.

HHs are mostly asymptomatic, solitary, and small in size and do not require further intervention or follow-up (3, 31). However, they also do not regress or disappear spontaneously, with possible expansion or dilation of the affected vessels during growth spurts and puberty (23). Follow up is suggested for patients with giant hemangiomas defined as tumors larger than 4 cm (28, 32). Our patient had multiple HHs, and the largest HH was 75 × 61 mm in size on US in March 2013, indicating that his HHs were giant HHs. The largest HH in the S6/7/8 liver segment was 86 × 81 × 81 mm in size on MRI in March 2014, suggesting that his HHs did not regress compared with that on US in March 2013. For patients with huge HHs or symptoms such as rapid tumor growth, persistent pain, pressure on adjacent organs or hemorrhage, treatment is recommended (28, 31, 32). Currently, the most preferred treatment methods, particularly for huge HHs, include surgical resection, transcatheter angiography embolization (TAE), radiofrequency ablation, radiotherapy or, in some cases, orthotopic liver transplantation (1, 31–33). However, the optimal surgical approach, either segmental resection or enucleation of the tumor based on the location of the lesion, remains controversial (33, 34). Transarterial embolization of the feeding artery and radiofrequency ablation have been suggested to induce a reduction in the size of a giant HH to reduce the risk of spontaneous or traumatic bleeding (33, 35). Radiotherapy is a less frequently used method for HH management, although it may have some effect on patients who cannot be treated with surgery because of the tumor size, multifocality, location, or patient condition and those who are not responding to drug therapy (36, 37). Liver transplantation is an important method of treatment for patients with a ruptured hemangioma (an extremely rare event, with an incidence of 1–4%) (8, 38). Although the risk of spontaneous or traumatic bleeding is very low, once it occurs and if emergency laparotomy cannot be performed in time, the mortality rate may be as high as 36–70% (12, 35). Although our patient had no discomfort during MRI examination, his HHs were so large that TAE was recommended to reduce the risk of a sudden rupture, which could lead to a poor prognosis. However, the patient rejected this suggestion. Although some previous studies have reported promising results with medical management of HHs using corticosteroids and beta-blockers, particularly propranolol, bevacizumab, sorafenib, interferon or a combination of sirolimus with high-dose propranolol (17–19, 21, 39), no definite drug is available to date that can be used to treat HH. In this patient, after taking various drugs to treat his secondary hypertension (including beta blockers effective for neonatal hemangioma) for 10 months, his multiple and giant HHs did not disappear or atrophy (Figure 2A). However, after adding AZM to treat tonsillitis and regulate his immunity for 6 months, the original small hemangiomas in the patient's liver disappeared, while the size of the large hemangiomas was significantly reduced (Figure 2B). After taking AZM for 11 months, most of the hemangiomas in the patient's liver disappeared, and the structure of the largest hemangioma was obscured with obvious calcification (Figure 1B). A review of the eating and living habits of the patient and his treatment strategies before and after the regression of his HHs found that the only change was the addition of AZM. Although no report has investigated the use of AZM to treat HHs, long-term low-dose treatment with AZM is associated with the regulation of MAPK signaling pathways [extracellular signal-regulated kinase (ERK) 1/2, p38^MAPK^, and c-Jun N-terminal kinase (JNK)] in respiratory diseases, including COPD, CF and non-cystic fibrobronchiectasis (40). AZM also effectively inhibits lung tumor growth by suppressing angiogenesis through its inhibition of VEGFR2-mediated downstream signaling pathways (27). Two major intracellular signaling pathways, the RAS/MAPK/ERK and phosphatidylinositol 3 kinase (PI3K)/protein kinase B (AKT)/mammalian target of rapamycin (mTOR) pathways, are implicated as the most important pathophysiological mechanisms for most vascular malformations (4, 24). HHs and infantile hemangioma (IH) are both congenital vascular anomalies (3, 16). VEGF is an important proangiogenic factor for endothelial cells, and anti-VEGF agents may be a valid treatment option for HHs (41). The above reports and effect of AZM on the HHs of this case indicate that AZM may be a drug treatment option for HHs.

## Conclusion

AZM exerts anti-inflammatory and immunomodulatory effects through the P38 MAPK, ERK1/2, and JNK pathways and plays an important role in the treatment of many respiratory diseases. Additionally, AZM inhibits tumor angiogenesis through VEGF-R-mediated downstream pathways. P38 MAPK-, ERK1-, and VEGF-related pathways are important regulatory targets for hemangioma, and anti-VEGF drugs affect IH. The patient's huge and multiple HHs significantly regressed after AZM treatment. Although this effect cannot be absolutely credited to AZM alone or a synergistic effect with other drugs (such as β-blockers), it may suggest that AZM is a potential drug treatment option for HHs. More importantly, this drug is very safe, even for pregnant women, infants or some patients with many underlying diseases. When a patient cannot tolerate surgery, the risk of surgery is too high, or other anti-hemangioma drugs have obvious side effects or are ineffective, AZM is a choice. Further clinical and experimental studies are warranted to determine the role and mechanism of AZM in HHs.

## Data Availability Statement

The raw data supporting the conclusions of this article will be made available by the authors, without undue reservation.

## Ethics Statement

All clinical data in this case report were either provided by the patient or collected by our team's members with their consent. There was no additional invasive test or experimental drugs used out of order for the patient. Written informed consent was obtained from the patient for the participation in the study and the publication of this report in accordance with the Declaration of Helsinki. The case report is exempt from institutional review board approval. Written informed consent was obtained from the individual(s) for the publication of any potentially identifiable images or data included in this article.

## Author Contributions

JL is the patient's family doctor, responsible for his long-term follow-up and writing of this article. JZ is responsible for collecting and analyzing all clinical data of the patient during his stay in the hospital where the JL is working. LT is responsible for the interpretation of all ultrasound results of the patient and the abdominal ultrasound follow-up in the hospital where the JL is working. ZY is responsible for collecting and analyzing all clinical data of the patient during his stay in the local hospital. All authors contributed to the article and approved the submitted version.

## Conflict of Interest

The authors declare that the research was conducted in the absence of any commercial or financial relationships that could be construed as a potential conflict of interest.

## Publisher's Note

All claims expressed in this article are solely those of the authors and do not necessarily represent those of their affiliated organizations, or those of the publisher, the editors and the reviewers. Any product that may be evaluated in this article, or claim that may be made by its manufacturer, is not guaranteed or endorsed by the publisher.

## References

[1] Hoekstra LT Bieze M Erdogan D Roelofs JJ Beuers UH van Gulik TM. Management of giant liver hemangiomas: an update. Expert Rev Gastroenterol Hepatol. (2013) 7:263–8. 10.1586/egh.13.10 23445235

[2] Hasan HY Hinshaw JL Borman EJ Gegios A Leverson G Winslow ER. Assessing normal growth of hepatic hemangiomas during long-term follow-up. JAMA Surg. (2014) 149:1266–71. 10.1001/jamasurg.2014.477 25321079

[3] Bajenaru N Balaban V Savulescu F Campeanu I Patrascu T. Hepatic hemangioma -review. J Med Life. (2015) 8:4–11. 26361504 PMC4564031

[4] Van Damme A Seront E Dekeuleneer V Boon LM Vikkula M. New and emerging targeted therapies for vascular malformations. Am J Clin Dermatol. (2020) 21:657–68. 10.1007/s40257-020-00528-w 32557381

[5] Mathew RP Sam M Raubenheimer M Patel V Low G. Hepatic hemangiomas: the various imaging avatars and its mimickers. Radiol Med. (2020) 125:801–15. 10.1007/s11547-020-01185-z 32249391

[6] Vilgrain V Boulos L Vullierme MP Denys A Terris B Menu Y. Imaging of atypical hemangiomas of the liver with pathologic correlation. Radiographics. (2000) 20:379–97. 10.1148/radiographics.20.2.g00mc01379 10715338

[7] Lewis S Aljarallah B Trivedi A Thung SN. Magnetic resonance imaging of a small vessel hepatic hemangioma in a cirrhotic patient with histopathologic correlation. Clin Imaging. (2015) 39:702–6. 10.1016/j.clinimag.2015.02.007 25748474

[8] Eghlimi H Arasteh P Azade N. Orthotopic liver transplantation for Management of a Giant Liver Hemangioma: a case report and review of literature. BMC Surg. (2020) 20:142. 10.1186/s12893-020-00801-z 32600292 PMC7324977

[9] Prodromidou A Machairas N Garoufalia Z Kostakis ID Tsaparas P Paspala A. Liver transplantation for giant hepatic hemangioma: a systematic review. Transplant Proc. (2019) 51:440–2. 10.1016/j.transproceed.2019.01.018 30879561

[10] Liu Y Wu X Ye L Xu H. Successful treatment of a patient with Kasabach-Merritt syndrome and multiple giant hepatic hemangiomas. J Int Med Res. (2020) 48:300060519898358. 10.1177/0300060519898358 31948308 PMC7113715

[11] Warren D Diaz L Levy M. Diffuse hepatic hemangiomas successfully treated using sirolimus and high-dose propranolol. Pediatr Dermatol. (2017) 34:e286–7. 10.1111/pde.13219 28730754

[12] Akhlaghpoor S Torkian P Golzarian J. Transarterial bleomycin-lipiodol embolization (B/LE) for symptomatic giant hepatic hemangioma. Cardiovasc Intervent Radiol. (2018) 41:1674–82. 10.1007/s00270-018-2010-4 29922860

[13] Cavalli R Novotna V Buffon RB Gelmetti C. Multiple cutaneous and hepatic infantile hemangiomas having a successful response to propranolol as monotherapy at neonatal period. G Ital Dermatol Venereol. (2013) 148:525–30. 24005146

[14] Satterfield KR Chambers CB. Current treatment and management of infantile hemangiomas. Surv Ophthalmol. (2019) 64:608–18. 10.1016/j.survophthal.2019.02.005 30772366

[15] Toro A Mahfouz AE Ardiri A Malaguarnera M Malaguarnera G Loria F. What is changing in indications and treatment of hepatic hemangiomas. A review. Ann Hepatol. (2014) 13:327–39. 10.1016/S1665-2681(19)30839-7 24927603

[16] Leon M Chavez L Surani S. Hepatic hemangioma: what internists need to know. World J Gastroenterol. (2020) 26:11–20. 10.3748/wjg.v26.i1.11 31933511 PMC6952297

[17] Greenberger S Boscolo E Adini I Mulliken JB Bischoff J. Corticosteroid suppression of VEGF-A in infantile hemangioma-derived stem cells. N Engl J Med. (2010) 362:1005–13. 10.1056/NEJMoa0903036 20237346 PMC2845924

[18] Boscolo E Mulliken JB Bischoff J. VEGFR-1 mediates endothelial differentiation and formation of blood vessels in a murine model of infantile hemangioma. Am J Pathol. (2011) 179:2266–77. 10.1016/j.ajpath.2011.07.040 21945324 PMC3204018

[19] Tiemann L Hein S. Infantile hemangioma: a review of current pharmacotherapy treatment and practice pearls. J Pediatr Pharmacol Ther. (2020) 25:586–99. 10.5863/1551-6776-25.7.586 33041713 PMC7541030

[20] Tang R Xian D Xu J Peng H Pan S Zhong J. Proanthocyanidins as a potential novel way for the treatment of hemangioma. Biomed Res Int. (2021) 2021:5695378. 10.1155/2021/5695378 33490272 PMC7801061

[21] Seront E Van Damme A Boon LM Vikkula M. Rapamycin and treatment of venous malformations. Curr Opin Hematol. (2019) 26:185–92. 10.1097/MOH.0000000000000498 30855337

[22] Adams DM Trenor CC III Hammill AM Vinks AA Patel MN Chaudry G. Efficacy and safety of sirolimus in the treatment of complicated vascular anomalies. Pediatrics. (2016) 137:e20153257. 10.1542/peds.2015-3257 26783326 PMC4732362

[23] Ricci KW. Advances in the medical management of vascular anomalies. Semin Intervent Radiol. (2017) 34:239–49. 10.1055/s-0037-1604297 28955113 PMC5615390

[24] Nguyen HL Boon LM Vikkula M. Vascular anomalies caused by abnormal signaling within endothelial cells: targets for novel therapies. Semin Intervent Radiol. (2017) 34:233–8. 10.1055/s-0037-1604296 28955112 PMC5615384

[25] Kitsiouli E Antoniou G Gotzou H Karagiannopoulos M Basagiannis D Christoforidis S. Effect of azithromycin on the LPS-induced production and secretion of phospholipase A2 in lung cells. Biochim Biophys Acta. (2015) 1852:1288–97. 10.1016/j.bbadis.2015.03.008 25791017

[26] Zhou X Zhang Y Li Y Hao X Liu X Wang Y. Azithromycin synergistically enhances anti-proliferative activity of vincristine in cervical and gastric cancer cells. Cancers (Basel). (2012) 4:1318–32. 10.3390/cancers4041318 24213508 PMC3712727

[27] Li F Huang J Ji D Meng Q Wang C Chen S. Azithromycin effectively inhibits tumor angiogenesis by suppressing vascular endothelial growth factor receptor 2-mediated signaling pathways in lung cancer. Oncol Lett. (2017) 14:89–96. 10.3892/ol.2017.6103 28693139 PMC5494938

[28] Stankiewicz R Kobryn K Patkowski W Krawczyk M. Management of giant hepatic hemangioma in atypical localization; report of a case and literature review. Pol Przegl Chir. (2015) 87:139–42. 10.1515/pjs-2015-0034 26146110

[29] Ito H Tsujimoto F Nakajima Y Igarashi G Okamura T Sakurai M. Sonographic characterization of 271 hepatic hemangiomas with typical appearance on CT imaging. J Med Ultrason (2001). (2012) 39:61–8. 10.1007/s10396-011-0339-2 27278845

[30] Mamone G Miraglia R. The “light bulb sign” in liver hemangioma. Abdom Radiol (NY). (2019) 44:2327–8. 10.1007/s00261-019-01964-x 31020349

[31] Ribeiro MA Jr Papaiordanou F Goncalves JM Chaib E. Spontaneous rupture of hepatic hemangiomas: a review of the literature. World J Hepatol. (2010) 2:428–33. 10.4254/wjh.v2.i12.428 21191518 PMC3010512

[32] Moctezuma-Velazquez C Lopez-Arce G Martinez-Rodriguez LA Escalona-Huerta C Chapa-Ibarguengoitia M Torre A. Giant hepatic hemangioma versus conventional hepatic hemangioma: clinical findings, risk factors, and management. Rev Gastroenterol Mex. (2014) 79:229–37. 10.1016/j.rgmxen.2014.12.004 25438870

[33] Jiang H Chen Z Prasoon P Wu H Zeng Y. Surgical management for giant liver hemangiomas greater than 20 cm in size. Gut Liver. (2011) 5:228–33. 10.5009/gnl.2011.5.2.228 21814606 PMC3140671

[34] Liu Y Wei X Wang K Shan Q Dai H Xie H. Enucleation versus anatomic resection for giant hepatic hemangioma: a meta-analysis. Gastrointest Tumors. (2017) 3:153–62. 10.1159/000455846 28611982 PMC5465724

[35] Sun JH Nie CH Zhang YL Zhou GH Ai J Zhou TY. Transcatheter arterial embolization alone for giant hepatic hemangioma. PLoS ONE. (2015) 10:e0135158. 10.1371/journal.pone.0135158 26287964 PMC4545419

[36] Kantor G Huchet A Remy S Pauillac M Mansir T Barrat P. Radiotherapy for a massive hepatic hemangioma in a six-week-old infant. Cancer Radiother. (1999) 3:503–7. 10.1016/S1278-3218(00)88258-X 10630164

[37] Gaspar L Mascarenhas F da Costa MS Dias JS Afonso JG Silvestre ME. Radiation therapy in the unresectable cavernous hemangioma of the liver. Radiother Oncol. (1993) 29:45–50. 10.1016/0167-8140(93)90172-5 8295987

[38] Cappellani A Zanghi A Di Vita M Zanghi G Tomarchio G Petrillo G: Spontaneous rupture of a giant hemangioma of the liver. Ann Ital Chir. (2000) 71:379–83. 11014019

[39] Greenberger S Bischoff J. Infantile hemangioma-mechanism(s) of drug action on a vascular tumor. Cold Spring Harb Perspect Med. (2011) 1:a006460. 10.1101/cshperspect.a006460 22229118 PMC3234458

[40] Yang J. Mechanism of azithromycin in airway diseases. J Int Med Res. (2020) 48:300060520932104. 10.1177/0300060520932104 32589092 PMC7323306

[41] Lee M Choi JY Lim JS Park MS Kim MJ Kim H. Lack of anti-tumor activity by anti-VEGF treatments in hepatic hemangiomas. Angiogenesis. (2016) 19:147–53. 10.1007/s10456-016-9494-9 26816001

